# Improvement of quality of life through glycemic control by liraglutide, a GLP-1 analog, in insulin-naive patients with type 2 diabetes mellitus: the PAGE1 study

**DOI:** 10.1186/s13098-016-0202-0

**Published:** 2017-01-07

**Authors:** Hitoshi Ishii, Tetsuji Niiya, Yasuhiro Ono, Naoyuki Inaba, Hideaki Jinnouchi, Hirotaka Watada

**Affiliations:** 1 Department of Diabetology, Nara Medical University, 840 Shijo-cho, Kashihara City, Nara, 634-8552 Japan; 2 Department of Internal Medicine, Matsuyama Shimin Hospital, Matsuyama, Ehime Japan; 3 Department of Medicine, Takagi Hospital, Okawa, Fukuoka, Japan; 4 Department of Metabolism & Endocrinology, Shizuoka Saiseikai General Hospital, Shizuoka, Japan; 5 Diabetes Care Center, Jinnouchi Hospital, Kumamoto, Japan; 6 Department of Metabolism & Endocrinology, Juntendo University Graduate School of Medicine, Tokyo, Japan

**Keywords:** Type 2 diabetes mellitus, T2DM, GLP-1, Liraglutide, Quality of life, DTR-QOL

## Abstract

**Background:**

In addition to achieving good glycemic control, diabetes care management aims to improve the quality of life (QOL) in patients. Treatment-associated difficulties and side effects frequently cause deterioration in QOL. Liraglutide, a GLP-1 receptor agonist, is a novel injection drug that promotes insulin secretion. It is a user-friendly, once-daily injection with fewer hypoglycemic events. In this study, we aimed to examine the effect of liraglutide therapy on QOL in patients.

**Methods:**

In total, 304 insulin- and liraglutide-naïve patients with type 2 diabetes were enrolled in this observational study; they received liraglutide therapy for 12 weeks. The main outcome measure was change in QOL from baseline, which was assessed using diabetes therapy-related QOL (DTR-QOL).

**Results:**

At week 12, liraglutide significantly decreased HbA1c levels (8.7 ± 1.5 vs. 7.5 ± 1.3, *p* < 0.001) and BMI (27.9 ± 5.3 vs. 27.3 ± 5.2, *p* < 0.001). According to the QOL scores, although the treatment modality had changed from non-injection to injection therapy, liraglutide improved patient satisfaction with treatment. Significant correlations were found between change in HbA1c level and satisfaction with treatment, as well as between change in body weight and burden on social and daily activities, anxiety and dissatisfaction with treatment, and hypoglycemia.

**Conclusions:**

Liraglutide significantly improved glycemic control and reduced the body weight without deteriorating QOL in obese patients with type 2 diabetes.

*Trial registration* UMIN-CTR: UMIN000007159

**Electronic supplementary material:**

The online version of this article (doi:10.1186/s13098-016-0202-0) contains supplementary material, which is available to authorized users.

## Background

The prevalence of type 2 diabetes mellitus (T2DM) has been increasing worldwide. Japan is one of the countries with the highest prevalence rates in the world [1], which was promoted by the adoption of high-fat westernized diet patterns and sedentary lifestyle due to the rise of automation. The persistent elevation of blood glucose level causes microvascular and life-threatening macrovascular complications resulting in low quality of life (QOL) in patients with diabetes.

Recent evidence suggests that good glycemic control is necessary to prevent diabetic complications. However, many patients with T2DM have difficulty achieving and maintaining glycemic control. One of the obstacles is that patients have to stick to a daily routine for a long period to maintain good glycemic control [2]. Another possible barrier includes the side effects of medical treatment including hypoglycemia or weight gain or both, which may arouse anxiety [3], decrease motivation, and lower the QOL [4] in patients. Patients often fail to adhere to suitable diabetes treatment because of these psychological stressors, which lead to a downward spiral of neglected diabetes care [5]. In particular, most patients have a negative attitude towards insulin injection [6], and consider it a critical-state treatment [7]. They become hesitant about the initiation of insulin therapy despite the fact that it is a reliable means to control blood glucose [8], which may result in late insulin initiation [9]. Thus, it is very important to attain good glycemic control without reducing patient motivation or QOL.

Liraglutide is a glucagon-like peptide-1 (GLP-1) receptor agonist. As GLP-1 receptor agonists promote insulin secretion in a blood glucose-dependent fashion, they cause fewer hypoglycemic episodes in comparison with sulfonylureas [10]. In addition, they can achieve long-term glycemic control with only one shot per day [11]. GLP-1 receptor agonists have other beneficial effects, such as suppression of appetite, delayed gastric emptying, and weight loss. These favorable features of liraglutide are expected to solve many of the unmet medical needs associated with T2DM treatment. However, it has been suggested that patients and physicians may be reluctant to implement liraglutide treatment because GLP-1 receptor agonists are injection drugs [4]. Therefore, it is important to elucidate the impact of liraglutide therapy on clinical parameters and QOL as well as its side effects in patients with T2DM.

We aimed to examine the effects of liraglutide on glycemic control, body weight, and QOL score in obese Japanese patients with T2DM in patient’s psychological attitude and glycemic control effectiveness by GLP-1 (PAGE1) study.

## Methods

### Research overview

We conducted a prospective, multicenter, pre-post observational study to examine the effect of liraglutide on QOL in Japanese patients with T2DM from February 2012 to September 2013 at 66 medical institutions in Japan listed in the Additional file 1. The inclusion criteria were (1) type 2 diabetes, (2) no prior use of insulin or liraglutide, and (3) aged 15 years and older. Patients with malignant tumors and pregnant or nursing women were excluded from this study. At the start of the study, only patients with dietary therapy, physical therapy, or sulfonylurea treatment were allowed to enroll because only they were allowed to use liraglutide under insurance coverage in Japan. During the course of the study, oral hypoglycemics other than sulfonylurea were reimbursed. Therefore, patients using those drugs were enrolled at later time points.

No pre-specified initiation or titration protocol for liraglutide was used. The participating physicians were allowed to determine the initial dose, maintenance dose, and timing of liraglutide administration by considering the patient’s condition and side effects. Clinical and laboratory parameters and diabetes therapy-related QOL (DTR-QOL) scores [12] were measured before (at baseline) and 12 weeks after the initiation of liraglutide therapy. In addition, at baseline and week 12, the hypoglycemic episodes that the patients experienced in the preceding four weeks were self-reported using the DTR-QOL questionnaire. The frequency of adverse events was evaluated to assess the safety of liraglutide therapy. The primary outcome measure was the change of DTR-QOL total score from baseline. Additionally, the correlation between changes in HbA1c and DTR-QOL scores was evaluated. Secondarily, correlations between changes in weight and DTR-QOL scores and those between changes in random blood glucose levels and DTR-QOL scores were evaluated.

The study protocol was registered with the University Hospital Medical Information Network (UMIN-CTR: UMIN000007159) prior to the commencement of the study. We adhered to the “Ethical Guidelines for Clinical Studies” issued by the Japanese government after receiving permission from the ethical committees at each of the participating medical facilities, and this study was conducted in accordance with the ethical standards laid down in the Declaration of Helsinki and its later amendments. All personal information was anonymized. The participation of patients with diabetes was obtained through an opt-out methodology. The patients were informed about the study and the ability to opt out via a poster. However, a written informed consent was given if directed by the institutional review board. To ensure data quality in this study, we contracted external entities for data collection, management, and statistical analysis.

### DTR-QOL questionnaire

We used the DTR-QOL questionnaire for evaluating QOL. The reliability and validity of the questionnaire was verified psychometrically [12], and it can be used to assess all modalities of diabetic treatment including injections. The self-administered questionnaire comprises 29 questions, and the patient answers each question using the 7-point Likert scale ranging from “Strongly disagree” (−7) to “Strongly agree” (1). The score of each item was reversed so that “7” represented the highest QOL. When calculating the scores for DTR-QOL questions 26–29, the rating scores were reversed such that a higher score indicated better QOL. The assessment covers each of the following four domains: D1 “Burden on social activities and daily activities,” D2 “Anxiety and dissatisfaction with treatment,” D3 “Hypoglycemia,” and D4 “Satisfaction with treatment.” By comparing the DTR-QOL scores before and after initiation of a new treatment, the influence and effects of the new treatment on patient QOL can be assessed quantitatively. The total score and domain scores were converted to a scale of 0–100 as described previously [12]. The patients filled out the DTR-QOL questionnaire by themselves in private, to avoid any influence of physicians and medical care providers.

### Statistical analysis

Patients were excluded from the analysis if data at baseline, week 12, or both time points were missing. We further excluded those who dropped out of the study or stopped liraglutide therapy owing to adverse effects. For the analysis of hypoglycemic events, we used all data, even when data for either of the two time points were missing. We calculated the total score and scores for each domain at both baseline and week 12, and compared them using a paired *t* test. All correlation analyses were performed using Spearman’s rank correlation coefficient. We conducted stratification analyses for DTR-QOL scores. DTR-QOL scores were compared using Student’s *t* test between the first and fourth groups stratified by quartiles of changes in HbA1c or body weight. The Wilcoxon signed-rank test was used to compare the scores of each question of the DTR-QOL questionnaire at baseline and week 12. The frequency of hypoglycemic events was compared between baseline and week 12 using the Chi squared test, and the number of hypoglycemic events per patient was compared using the Wilcoxon rank-sum test. A paired *t* test was used for comparing the clinical and biochemical characteristics at both time points.

Based on a previous study [12], power analysis was performed to detect a change of 7 points in the DTR-QOL total score (10% improvement), with two-sided alpha of 5% and beta of 7.5%. Assuming a DTR-QOL total score at baseline ±SD of 70 ± 15 and a dropout rate of 30%, we calculated that at least 300 patients were needed for this study. All statistical tests were two-sided with an alpha level of 0.05 and performed using the SAS 9.3 software.

## Results

### Subject characteristics

**Fig. 1:**
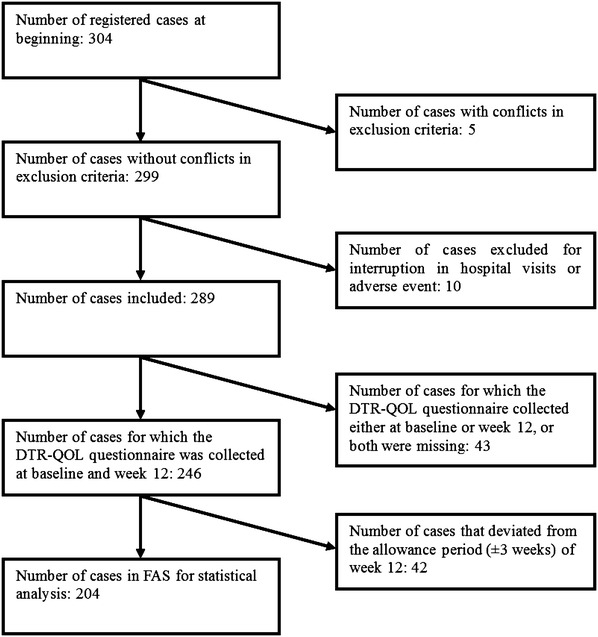
Flowchart of selection of patients with T2DM for the analysis

In total, 304 Japanese patients were enrolled in this study. After enrollment, five cases were found to meet the exclusion criteria, ten cases dropped out, 43 cases did not have questionnaire data either at baseline or week 12, and 42 cases deviated from the allowance period at week 12. Thus, we used data from 204 cases for analyses (Fig. 1). The ten dropouts included three patients who stopped hospital visits and seven who dropped out because of adverse events: four cases presented gastrointestinal symptoms (constipation, loss of appetite, gastritis, and bloating), one case showed hypersensitivity (hives), one showed hyperglycemia, and one showed depression. No dropouts due to hypoglycemia were noted. The mean age was 59.4 ± 12.3 years, mean weight was 73.9 ± 17.0 kg, mean BMI (body mass index) was 27.9 ± 5.3 kg/m^2^, mean HbA1c was 8.7 ± 1.5% (NGSP, National Glycohemoglobin Standardization Program; 71.6 ± 16.7 mmol/mol), and the mean duration of diabetes was 115.0 ± 88.8 months (9.6 ± 7.4 years). In total, 55.9% of subjects used combination therapy with sulfonylurea during this study (Table 1). The concomitant drugs used by these patients at baseline and week 12 are shown in Table 2, which indicated that their medication was not changed within 12 weeks. Concerning the dose of liraglutide, 91.8% of the patients started 0.3 mg/day at baseline, and 77.0% of the patients received 0.9 mg/day after 12 weeks (Table 3).

**Table 1:** Patient characteristics at baseline

Characteristics	Value
Age, years	59.4 ± 12.3
Height, cm	162.6 ± 9.6
Duration of diabetes, months	115.0 ± 88.8
Men	119 (58.3)
Macrovascular complications of diabetes	36 (17.7)
Arteriosclerosis obliterans	8 (3.9)
Coronary heart disease	19 (9.3)
Stroke	15 (7.3)
Diabetic microangiopathy	106 (52.0)
Diabetic retinopathy	49 (24.0)
Diabetic neuropathy	47 (23.0)
Diabetic nephropathy	83 (40.6)
Sulfonylurea before using GLP-1	123 (60.3)

*n* = 204 except for height (*n* = 203) and duration of diabetes (*n* = 197) because of missing data. Data are shown as the mean ± SD or the number of patients (%)

**Table 2:** Concomitant drugs

Drugs	Baseline (*n* = 204)	Week 12 (*n* = 204)
Antidiabetic drug	140 (68.6)	138 (67.7)
Sulfonylurea	114 (55.9)	113 (55.4)
α-Glucosidase inhibitor	19 (9.3)	18 (8.8)
Biguanide	47 (23.0)	49 (24.0)
DPP-4 inhibitor	8 (3.9)	7 (3.4)
Glinide	3 (1.5)	3 (1.5)
Thiazolidinedione	10 (4.9)	10 (4.9)
Other	1 (0.5)	0 (0.0)
Antihypertensive drug	121 (59.3)	123 (60.3)
Diuretic drug	22 (10.8)	20 (9.8)
Calcium channel blocker	75 (36.8)	77 (37.8)
ACE inhibitor	9 (4.4)	8 (3.9)
Angiotensin II receptor blocker	95 (46.6)	98 (48.0)
Direct renin inhibitor	0 (0.0)	0 (0.0)
α-blocker	0 (0.0)	0 (0.0)
β-blocker	17 (8.3)	18 (8.8)
α1β-blocker	4 (2.0)	5 (2.5)
α2 receptor agonist	1 (0.5)	1 (0.5)
Other	2 (1.0)	2 (1.0)
Lipid-lowering agent	117 (57.4)	118 (57.8)
Statin	103 (50.5)	102 (50.0)
Fibrate	12 (5.9)	12 (5.9)
Ezetimibe	6 (2.9)	8 (3.9)
Probucol	0 (0.0)	0 (0.0)
EPA	0 (0.0)	0 (0.0)
Resin	0 (0.0)	0 (0.0)
Other	4 (2.0)	4 (2.0)

Data are shown as *n* (%)

*DPP*-*4* dipeptidyl peptidase-4, *ACE* angiotensin-converting enzyme, *EPA* eicosapentaenoic acid

**Table 3:** Liraglutide dose

Dose (mg)	Baseline (*n* = 204)	Week 12 (*n* = 204)
0.3	186 (91.2)	19 (9.3)
0.6	3 (1.5)	28 (13.7)
0.9	15 (7.4)	157 (77.0)

Data are shown as *n* (%)

### Effects of liraglutide on clinical and biochemical parameters, and incidence of hypoglycemia

The clinical and biochemical parameters at the two evaluated time points are shown in Table 4. The HbA1c change was −1.2 ± 0.1% (−13.0 ± 1.2 mmol/mol), demonstrating a significant improvement in glycemic control (*p* < 0.001). Body weight and BMI were significantly decreased (*p* < 0.001 for both) by −1.4 ± 0.3 kg and −0.5 ± 0.1 kg/m^2^, respectively. Significant decreases were also observed in total cholesterol (−8.4 ± 2.8 mg/dL [−0.2 ± 0.07 mmol], *p* = 0.003), uric acid (−0.2 ± 0.1 mg/dL [−11.9 ± 5.9 mmol], *p* = 0.025), and HDL-C (−1.2 ± 0.6 mg/dL [−0.03 ± 0.02 mmol], *p* = 0.041).

**Table 4:** Clinical and biochemical parameters at baseline and at week 12 of liraglutide treatment

Items	Mean value ± SD		*n*	Change (SE)	*p* value
	Baseline	Week 12			
Weight (kg)	73.9 ± 17.0	72.5 ± 16.7	204	−1.4 (0.3)	<0.001
BMI (kg/m^2^)	27.9 ± 5.3	27.3 ± 5.2	203	−0.5 (0.1)	<0.001
Systolic blood pressure (mmHg)	131.1 ± 15.4	129.4 ± 16.0	203	−1.8 (1.2)	0.134
Diastolic blood pressure (mmHg)	76.0 ± 11.6	76.4 ± 12.2	203	0.4 (0.8)	0.652
HbA1c (NGSP, %)	8.7 ± 1.5	7.5 ± 1.3	203	−1.2 (0.1)	<0.001
Random blood glucose level (mg/dL)	199 ± 80	170 ± 66	198	−29.0 (5.8)	<0.001
RBCs (×10^4^/μL)	459 ± 54	458 ± 50	135	−1.1 (2.5)	0.661
WBCs (/mm^3^)	6887 ± 1879	6913 ± 1876	135	26.4 (140.4)	0.851
Hemoglobin (g/dL)	13.9 ± 1.6	13.9 ± 1.5	135	0.0 (0.1)	0.886
Hematocrit (%)	41.6 ± 4.2	41.8 ± 4.2	135	0.2 (0.2)	0.225
Blood platelets (×10^4^/μL)	22.5 ± 6.2	23.1 ± 5.7	134	0.6 (0.4)	0.088
AST (IU/L)	28.1 ± 20.6	26.6 ± 19.3	162	−1.5 (1.1)	0.176
ALT (IU/L)	36.1 ± 33.1	33.5 ± 29.0	160	−2.7 (1.6)	0.090
γGTP (IU/L)	50.0 ± 52.7	50.2 ± 64.8	153	0.2 (2.9)	0.943
Serum creatinine (mg/dL)	0.9 ± 1.0	0.9 ± 0.9	162	0.0 (0.0)	0.215
Uric acid (mg/dL)	5.6 ± 1.5	5.4 ± 1.4	151	−0.2 (0.1)	0.025
TC (mg/dL)	189.1 ± 36.5	180.8 ± 32.2	117	−8.4 (2.8)	0.003
HDL-C (mg/dL)	50.9 ± 12.9	49.7 ± 12.5	155	−1.2 (0.6)	0.041
TG (mg/dL)	177.9 ± 130.3	172.3 ± 100.8	166	−5.6 (7.4)	0.451

*BL* baseline, *w12* week 12, *RBCs* red blood cells, WBCs white blood cells, *BMI* body mass index *HbA1c* hemoglobin A1c, *NGSP* National Glycohemoglobin Standardization Program, *AST* aspartate aminotransferase, *ALT* alanine aminotransferase, *γGTP* γ-glutamyl transpeptidase, *TC* total cholesterol, *HDL*-*C* high density lipoprotein cholesterol, *TG* triglyceride

*p* values are the results of paired *t* test

The proportion of patients experiencing hypoglycemic events during the 4 weeks prior to baseline and week 12 were 13.8 and 15.5% (*p* = 0.758), respectively. The number of hypoglycemic events per patient at both time points was 8.1 ± 8.5 and 5.3 ± 7.4 (*p* = 0.150), respectively (Additional file 2: Figure S1).

### Effects of liraglutide on DTR-QOL scores

Changes in DTR-QOL total score and each of the four domain scores are shown in Table 5. The total score of 198 subjects at baseline was 61.9 ± 16.2, and it was significantly improved to 69.7 ± 16.8 (*p* < 0.001) at week 12. Significant improvement was also seen in all four domains (D1–D4; Table 5). The effect size [13] for the total score was 0.48. The effect sizes for the scores in domains D1, D2, D3, and D4 were 0.28, 0.48, 0.23, and 0.61, respectively. The effect size was the largest for D4, followed by total score and D2, which reflected moderate effect on QOL [14]. When assessing the effect for each individual question of the questionnaire, a significant improvement was observed for 24 of 29 questions (Table 6). However, significant decreases were found in the scores for Q12, “Pain due to my current diabetes treatment is uncomfortable,” and Q13, “Gastrointestinal symptoms (nausea, passing gas, diarrhea, abdominal pain) due to my current diabetes treatment are uncomfortable.” No significant changes were found for the following three questions: Q5, “It is a burden getting up at a certain time every morning for my current diabetes treatment,” Q15, “I worry about low blood glucose due to my current diabetes treatment,” and Q25, “I am concerned that if I continue my current diabetes treatment, the efficacy (effectiveness) may diminish.”

**Table 5:** DTR-QOL total and domain scores

Domain		Score		*n*	*p* value	Effect siz*e*
		Baseline	Week 12			
D1:	Burden on social activities and daily activities	68.2 ± 20.2	73.9 ± 19.8	204	<0.001	0.28
D2:	Anxiety and dissatisfaction with treatment	51.1 ± 21.9	61.5 ± 21.9	201	<0.001	0.48
D3:	Hypoglycemia	75.4 ± 26.0	81.5 ± 23.2	200	0.002	0.23
D4:	Satisfaction with treatment	48.3 ± 21.5	61.4 ± 21.3	202	<0.001	0.61
Total score		61.9 ± 16.2	69.7 ± 16.8	198	<0.001	0.48

The total score and domain scores were converted to a scale of 0–100

*p* values are the results of paired *t* test

**Table 6:** Results of DTR-QOL questionnaire

Domain	Questions		Score		*n*	*p* value
			Baseline	Week 12		
D1: burden on social activities and daily activities	Q1	My current diabetes treatment interferes with my work and activities	4.9 ± 2.0	5.5 ± 1.8	204	<0.001
	Q2	My current diabetes treatment limits the scope of my activities	5.1 ± 2.0	5.6 ± 1.7	203	<0.001
	Q3	It is difficult to find places on time for my current diabetes treatment	5.4 ± 1.7	5.8 ± 1.6	203	0.001
	Q4	My current diabetes treatment interferes with group activities and personal friendships	5.4 ± 1.8	5.8 ± 1.7	204	0.007
	Q5	It is a burden getting up at a certain time every morning for my current diabetes treatment	5.8 ± 1.7	5.9 ± 1.5	204	0.151
	Q6	With my current diabetes treatment, the restricted meal times are a burden	5.1 ± 1.9	5.7 ± 1.6	204	<0.001
	Q7	When I eat out, it is difficult to manage my current diabetes treatment	4.8 ± 1.9	5.4 ± 1.8	203	<0.001
	Q8	I feel like my current diabetes treatment takes away the enjoyment of eating	4.3 ± 1.9	4.8 ± 1.9	204	<0.001
	Q9	With my current diabetes treatment, it is hard to curb my appetite	3.8 ± 2.0	4.6 ± 1.9	204	<0.001
	Q10	The time and effort to manage my current diabetes treatment are a burden	5.0 ± 1.7	5.3 ± 1.7	204	0.018
	Q11	I am constantly concerned about time to manage my current diabetes treatment	5.2 ± 1.7	5.6 ± 1.6	204	0.002
	Q12	Pain due to my current diabetes treatment is uncomfortable	5.9 ± 1.6	5.4 ± 1.7	204	<0.001
	Q13	Gastrointestinal symptoms (nausea, passing gas, diarrhea, abdominal pain) due to my current diabetes treatment are uncomfortable	5.7 ± 1.7	5.2 ± 1.9	204	0.001
D2: anxiety and dissatisfaction with treatment	Q14	I am bothered by weight gain with my current diabetes treatment	4.7 ± 2.0	5.6 ± 1.7	200	<0.001
	Q19	I have uncomfortable symptoms due to hyperglycemia (high blood glucose)	5.0 ± 2.0	5.4 ± 1.7	200	0.012
	Q20	I am worried about high blood glucose	3.5 ± 2.0	4.6 ± 2.0	199	<0.001
	Q21	I am dissatisfied that my blood glucose is unstable (high and low)	3.9 ± 1.9	4.7 ± 1.7	201	<0.001
	Q22	I am worried that complications might get worse with my current diabetes treatment	3.3 ± 2.0	4.1 ± 2.0	201	<0.001
	Q23	I get anxious thinking about living while on my current diabetes treatment	3.9 ± 2.0	4.4 ± 1.9	201	0.001
	Q24	I find it unbearable to think that even if I continue my current diabetes treatment, my diabetes may not be cured	3.9 ± 1.9	4.3 ± 1.8	203	<0.001
	Q25	I am concerned that if I continue my current diabetes treatment, the efficacy may diminish	4.5 ± 1.8	4.5 ± 1.8	203	0.822
D3: hypoglycemia	Q15	I worry about low blood glucose due to my current diabetes treatment	5.3 ± 1.8	5.5 ± 1.6	201	0.100
	Q16	I am scared because of low blood glucose	5.6 ± 1.7	6.0 ± 1.5	200	<0.001
	Q17	I am sometimes bothered by low blood glucose	5.7 ± 1.7	6.1 ± 1.4	200	0.001
	Q18	Symptoms due to low blood glucose are uncomfortable	5.6 ± 1.8	5.9 ± 1.6	199	0.002
D4: satisfaction with treatment	Q26	Overall, I am satisfied with my current blood sugar control	3.2 ± 1.7	4.3 ± 1.8	202	<0.001
	Q27	With my current diabetes treatment, I am confident that I can maintain good blood glucose control	3.7 ± 1.6	4.6 ± 1.5	202	<0.001
	Q28	I am hopeful about the future with my current diabetes treatment	4.5 ± 1.5	4.9 ± 1.4	204	0.001
	Q29	With regards to diabetes treatment, I am satisfied with current treatment methods	4.1 ± 1.5	5.0 ± 1.4	204	<0.001

Scores are shown as the mean ± SD

“7” represents the highest QOL score of each item

*p* values are the results of the Wilcoxon signed-rank test

### Correlations between changes in DTR-QOL scores and changes in HbA1c, body weight, and random blood glucose

**Fig. 2:**
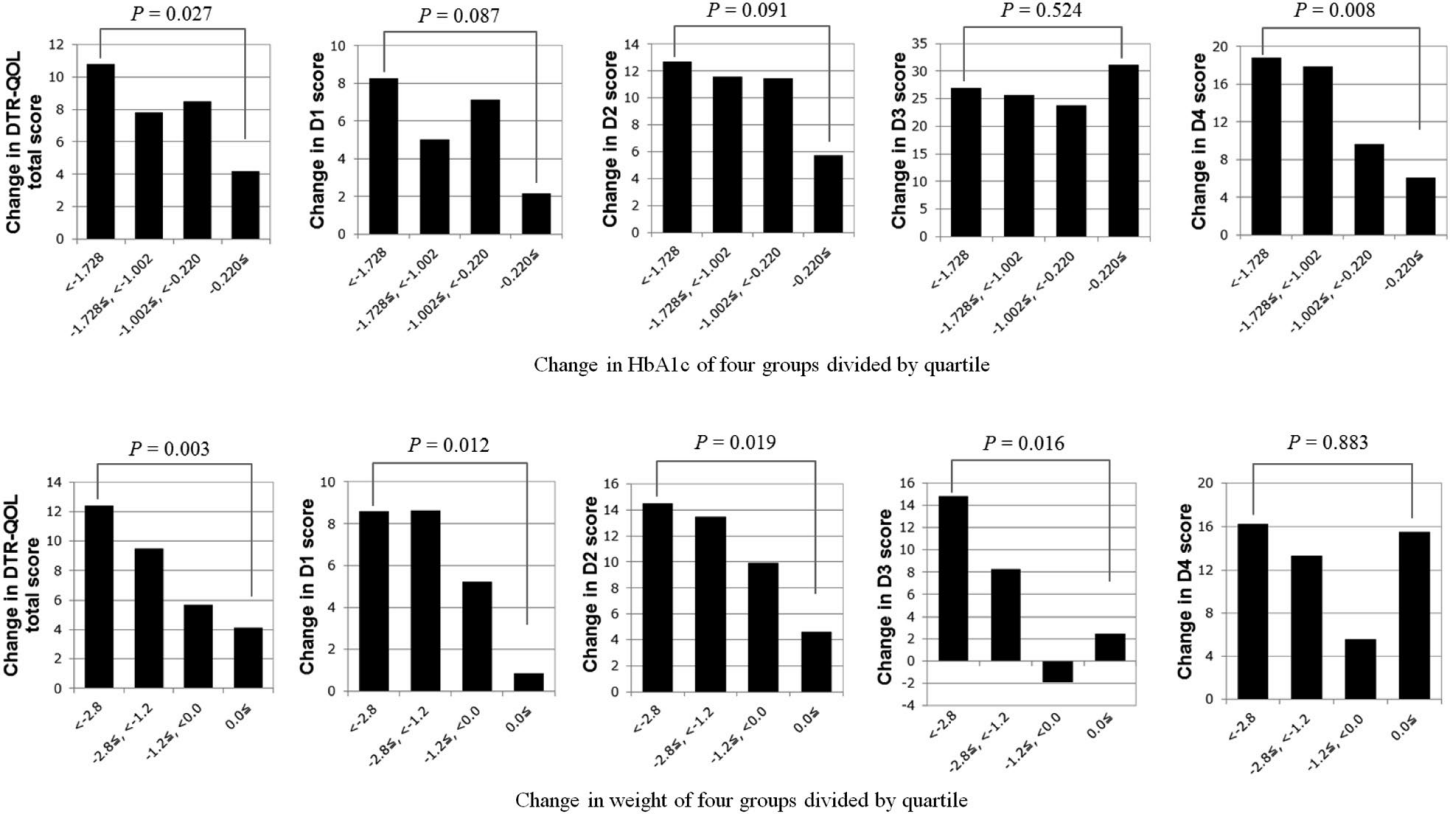
Changes of DTR-QOL scores in each quartile of the change of HbA1c or body weight. The changes in the total DTR-QOL score and each domain score between baseline and 12 weeks after the initiation of liraglutide in each quartile of change in HbA1c and body weight are shown

We found a significant correlation between change in DTR-QOL total score and change in body weight (*ρ* = −0.24, *p* < 0.001), but not with change in HbA1c (*ρ* = −0.12, *p* = 0.102) and random blood glucose (*ρ* = 0.03, *p* = 0.702). With regard to changes in the four DTR-QOL domain scores, we detected significant correlations for the following parameters: HbA1c and D4 (*ρ* = −0.22, *p* = 0.002), and weight and D1 (*ρ* = −0.18, *p* = 0.010), D2 (*ρ* = −0.20, *p* = 0.006), and D3 (*ρ* = −0.22, *p* = 0.002; Additional file 3: Table S1). Stratification by HbA1c revealed significant changes between the first and fourth quartiles not only in D4 score (*p* = 0.008), but also in the total score (*p* = 0.027; Fig. 2). Similarly, significant differences were found in total, D1, D2, and D3 scores between the groups of the first and fourth quartiles of weight change (*p* = 0.003, 0.012, 0.019, 0.016, respectively).

## Discussion

In this study, we showed the efficacy of liraglutide, and its influence on changes in DTR-QOL scores, and the correlations between changes in HbA1c/body weight/random blood glucose levels and DTR-QOL scores in T2DM patients who did not have prior experience with injection therapy. Liraglutide therapy decreased HbA1c level and body weights, and improved patient QOL as evidenced by the increase in scores for 24 of 29 questions, covering more than 82% of the DTR-QOL questionnaire.

When compared to patients who planned to start first-time insulin therapy [15] or those using oral hypoglycemic agents (OHA) alone [16], the following features were observed in this cohort: better glycemic control than patients considering insulin injections [15], but not better than those using OHA alone [16]; younger; shorter duration of T2DM; and higher BMI [19]. With regard to high BMI, obese T2DM patients with poor glycemic control in OHA therapy seemed to have been selected as candidates for this study in the hope of weight loss via liraglutide [17]. As expected, we observed a significant reduction in weight, BMI, and HbA1c at 12 weeks after liraglutide initiation. Notably, all four domains in DTR-QOL as well as the total score improved, which was rather unexpected because once-daily self-injection of liraglutide was supposed to pose a burden for patients or negatively influence their QOL [18]. We speculate that the improvement in clinical parameters by the liraglutide treatment changed the patients’ perception of T2DM treatment from negative to positive, even though they required additional self-injection of liraglutide, as is clear from Table 4 and Fig. 2. In other words, the difficulties of daily medication and additional liraglutide injection were overruled by the satisfaction associated with the beneficial outcomes generated by liraglutide. Another possibility is that patients felt that injection therapy turned out easier than expected. However, patients reported decreased QOL concerning side effects of liraglutide injection such as pain due to self-injection (Q12) and gastrointestinal symptoms (Q13). These results seem logical because gastrointestinal symptoms are well-known side effects of the drug, and the patients enrolled in our study were first-time users of self-injection therapy. Accordingly, we believe that the results of the DTR-QOL questionnaire represent not only subjective reality, but also objective reality in patients who started liraglutide treatment.

Among the four domains of DTR-QOL, marked improvements were observed for D2 (anxiety and dissatisfaction with treatment) and D4 (satisfaction with treatment), reflecting the achievement of satisfaction with improved clinical parameters. In particular, significantly improved scores were confirmed for all four questions in D4, indicating that patients with liraglutide treatment were satisfied and confident about glycemic control. A significant correlation was observed between the change in HbA1c and D4 score (*ρ* = −0.22, *p* = 0.002). Similarly, previous studies have demonstrated a correlation between changes in QOL score and HbA1c [19], with similar correlation coefficients [20, 21]. Furthermore, quartile-stratified analysis showed a large difference between groups of the first and fourth quartiles of change in HbA1c (Fig. 2), suggesting an association between the change of HbA1c and D4.

The combination of liraglutide therapy with OHA treatment was expected to increase the risk and fear of hypoglycemic events. In contrast, it resulted in the improvement of D3 (hypoglycemia). However, the number of patients who experienced hypoglycemic events, and the number of hypoglycemic events per patient did not change after the initiation of liraglutide therapy. We consider that the increase in D3 score was because of the improvement in clinical parameters without a rise in hypoglycemic events.

Weight change was negatively correlated with changes in the DTR-QOL total score and D1–D3, indicating that the greater the weight loss, the higher the QOL. Changes in these scores were as high as 8.6–14.8 in the first quartile groups of body weight change and as low as 0.8–4.6 in the fourth quartile groups (Fig. 2), strongly supporting the association between increase of QOL and body weight reduction.

There are several limitations to this study. First, it was a single-arm, pre-post observational study without control arm. Owing to this limitation, we do not know whether the results obtained include a placebo effect. A subset of patients might lose weight in response to the self-injection of placebo, and weight loss alone (independent of treatment) could provide combined improvement in HbA1c and QOL indicators seen in the study. Second, we excluded the data of 43 patients for whom we did not have data at both baseline and week 12. Therefore, it is difficult to extrapolate the findings of this study to all T2DM patients. Nonetheless, this study demonstrated that the injection of liraglutide along with OHA treatment reduced the body weight, BMI, and HbA1c level, and improved QOL, except for the gastrointestinal symptoms and pain associated with liraglutide injection, in obese T2DM patients. Physicians sometimes hesitate to initiate the use of injection therapies. However, this study revealed that self-injection does not necessarily deteriorate the patient’s QOL. Based on the routine subjective assessment of patient QOL, patients and physicians can select better treatment options.

## Conclusions

In conclusion, liraglutide is an effective treatment option for obese T2DM patients, and it helps in reducing body weight and improving glycemic control without deteriorating QOL.

## Additional files

**Additional file 1.** List of medical facilities.

**Additional file 2: Figure S1.** Figure showing hypoglycemic events before and after liraglutide treatment.

**Additional file 3: Table S1.** Table describing the correlation between domain scores and HbA1c, body weight, and random blood glucose.

## Abbreviations

QOL: quality of life
DTR-QOL: diabetes therapy-related QOL
BMI: body mass index
T2DM: type 2 diabetes mellitus
NGSP: National Glycohemoglobin Standardization Program
