# Supplementary material for: Improvement of quality of life through glycemic control by liraglutide, a GLP-1 analog, in insulin-naive patients with type 2 diabetes mellitus: the PAGE1 study
Source: Diabetol Metab Syndr. 2017 Jan 7;9:3. doi: 10.1186/s13098-016-0202-0 (PMC5219656; doi:10.1186/s13098-016-0202-0)
Supplement: Supplementary file 1 — Additional file 1. List of medical facilities. [file 13098_2016_202_MOESM1_ESM.docx]

**Additional file 1.**

**Caption:** List of medical facilities

**Title:**

Improvement of quality of life through glycemic control by liraglutide, a GLP-1 analog, in insulin-naive patients with type 2 diabetes mellitus: the PAGE1 study

Hitoshi Ishii^1^, Tetsuji Niiya^2^, Yasuhiro Ono^3^, Naoyuki Inaba^4^, Hideaki Jinnouchi^5^, Hirotaka Watada^6^

^1^ Department of Diabetology, Nara Medical University, Kashihara, Japan

^2^ Department of Internal Medicine, Matsuyama Shimin Hospital, Matsuyama, Ehime, Japan

^3^ Department of Medicine, Takagi Hospital, Okawa, Fukuoka, Japan

^4^ Department of Metabolism & Endocrinology, Shizuoka Saiseikai General Hospital, Shizuoka, Japan

^5^ Diabetes Care Center, Jinnouchi Hospital, Kumamoto, Japan

^6^ Department of Metabolism & Endocrinology, Juntendo University Graduate School of Medicine, Tokyo, Japan

**Corresponding author:** Hitoshi Ishii, Department of Diabetology, Nara Medical University, 840 Shijo-cho, Kashihara City, Nara, 634-8552, Japan.

Tel: +81-744-22-3051; Fax: +81-744-29-8811; E-mail: hit3910@gmail.com

**List of 66 medical institutions participating in the study.**

Yao Tokushukai General Hospital, Matsuyama Shimin Hospital, Takagi Hospital, Shizuoka Saiseikai General Hospital, Nishiohmiya Hospital, Juntendo University Hospital, Kanto Rosai Hospital, Juntendo University Shizuoka Hospital, St. Mary's Hospital, Manda Memorial Hospital, Nakayama Clinic, Takahashi Family Clinic, Yayoi Medical Clinic, Yokohama Rosai Hospital, Kushiro Red Cross Hospital, Kitaakita Municipal Hospital, Seino Internal Medicine Clinic, Green Clinic, Sakashita Hospital, Nerima Daiichi Shinryoujo, Kumanomae Nishimura Clinic, Senpo Tokyo Takanawa Hospital, Tokyo Women's Medical University Hospital Metabolism and Diabetology, Nagaoka Chuo General Hospital, Wakayama Rosai Hospital, National Hospital Organization Minami-Okayama Medical Center, The Sakakibara Heart Institute of Okayama, Iwamoto Naika Iin, Chiba Iin, Azusawa Hospital, Suruga Clinic, Toyonaka-Wakabakai Hospital, Jinnouchi Hospital, Minami Akatsuka Clinic, Sanshikai Toho Hospital, Mirai Clinic, Japanese Red Cross Medical Center, Fukuda Clinic, Yoshinogawa Medical Center, Dojinkai Yoshida Hospital, Kiryu Kosei General Hospital, Yokohama Sakae Kyosai Hospital, Sone Hospital, Kyoujinkai Komatsu Hospital, Takekawa Clinic, Osaka Rosai Hospital, Ikeda City Hospital, Shimizu Clinic, Yao Municipal Hospital, Tenri Hospital, Tokushima Red Cross Hospital, Okamoto Naika Clinic, Sapporo City General Hospital, Fukushima Medical University, Ozawa Hospital, Minagawa Clinic, Nagaoka Red Cross Hospital, Hamamatsu Medical Center, Seishinkai Okamoto Naika Iin, Ikeura Clinic, Takagi Naika Clinic, Mimihara Oimatsu Clinic, Ikeda Shinryoujyo, Tokushima University Hospital, Okada Clinic, Tamana Central Hospital.
