# Supplementary material for: Improvement of quality of life through glycemic control by liraglutide, a GLP-1 analog, in insulin-naive patients with type 2 diabetes mellitus: the PAGE1 study
Source: Diabetol Metab Syndr. 2017 Jan 7;9:3. doi: 10.1186/s13098-016-0202-0 (PMC5219656; doi:10.1186/s13098-016-0202-0)

**Additional file 2.**

**Caption:** Figure showing hypoglycemic events before and after liraglutide treatment

**Title:**

Improvement of quality of life through glycemic control by liraglutide, a GLP-1 analog, in insulin-naive patients with type 2 diabetes mellitus: the PAGE1 study

Hitoshi Ishii^1^, Tetsuji Niiya^2^, Yasuhiro Ono^3^, Naoyuki Inaba^4^, Hideaki Jinnouchi^5^, Hirotaka Watada^6^

^1^ Department of Diabetology, Nara Medical University, Kashihara, Japan

^2^ Department of Internal Medicine, Matsuyama Shimin Hospital, Matsuyama, Ehime, Japan

^3^ Department of Medicine, Takagi Hospital, Okawa, Fukuoka, Japan

^4^ Department of Metabolism & Endocrinology, Shizuoka Saiseikai General Hospital, Shizuoka, Japan

^5^ Diabetes Care Center, Jinnouchi Hospital, Kumamoto, Japan

^6^ Department of Metabolism & Endocrinology, Juntendo University Graduate School of Medicine, Tokyo, Japan

**Corresponding author:** Hitoshi Ishii, Department of Diabetology, Nara Medical University, 840 Shijo-cho, Kashihara City, Nara, 634-8552, Japan.

Tel: +81-744-22-3051; Fax: +81-744-29-8811; E-mail: hit3910@gmail.com

**Figure S1. Effects of liraglutide on hypoglycemic events. (A)** The proportion of patients experiencing hypoglycemic events during the 4 weeks prior to baseline and week 12 after the initiation of liraglutide treatment. **(B)** The number of hypoglycemic events during the 4 weeks prior to baseline and week 12 after the initiation of liraglutide treatment. Data in (B) are represented as the means ± SD


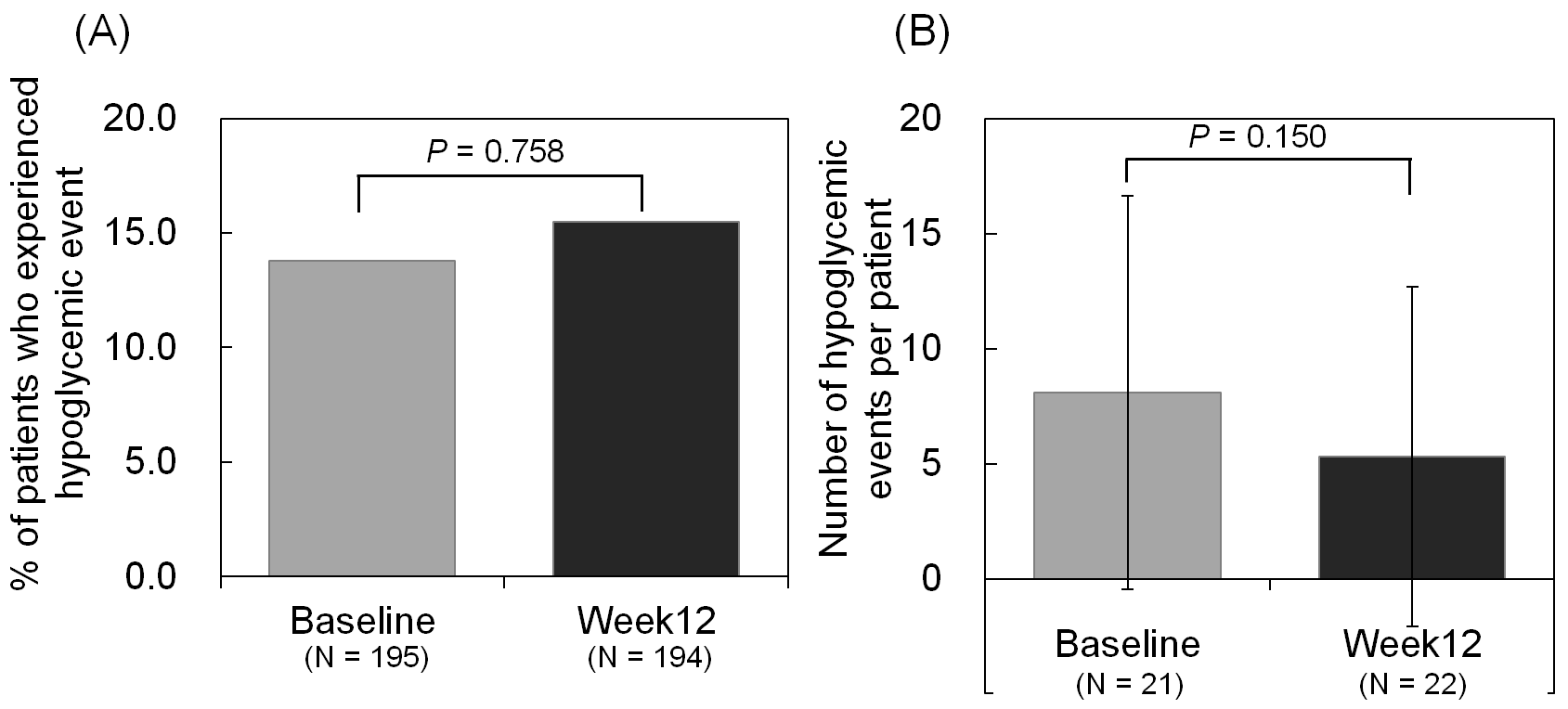

Supplement: Supplementary file 2 — Additional file 2: Figure S1. Figure showing hypoglycemic events before and after liraglutide treatment. [file 13098_2016_202_MOESM2_ESM.docx]
