# Supplementary material for: Improvement of quality of life through glycemic control by liraglutide, a GLP-1 analog, in insulin-naive patients with type 2 diabetes mellitus: the PAGE1 study
Source: Diabetol Metab Syndr. 2017 Jan 7;9:3. doi: 10.1186/s13098-016-0202-0 (PMC5219656; doi:10.1186/s13098-016-0202-0)
Supplement: Supplementary file 3 — Additional file 3: Table S1. Table describing the correlation between domain scores and HbA1c, body weight, and random blood glucose. [file 13098_2016_202_MOESM3_ESM.docx]

**Additional file 3.**

**Caption:** Table describing the correlation between domain scores and HbA1c, body weight, and random blood glucose

**Title:**

Improvement of quality of life through glycemic control by liraglutide, a GLP-1 analog, in insulin-naive patients with type 2 diabetes mellitus: the PAGE1 study

Hitoshi Ishii^1^, Tetsuji Niiya^2^, Yasuhiro Ono^3^, Naoyuki Inaba^4^, Hideaki Jinnouchi^5^, Hirotaka Watada^6^

^1^ Department of Diabetology, Nara Medical University, Kashihara, Japan

^2^ Department of Internal Medicine, Matsuyama Shimin Hospital, Matsuyama, Ehime, Japan

^3^ Department of Medicine, Takagi Hospital, Okawa, Fukuoka, Japan

^4^ Department of Metabolism & Endocrinology, Shizuoka Saiseikai General Hospital, Shizuoka, Japan

^5^ Diabetes Care Center, Jinnouchi Hospital, Kumamoto, Japan

^6^ Department of Metabolism & Endocrinology, Juntendo University Graduate School of Medicine, Tokyo, Japan

**Corresponding author:** Hitoshi Ishii, Department of Diabetology, Nara Medical University, 840 Shijo-cho, Kashihara City, Nara, 634-8552, Japan.

Tel: +81-744-22-3051; Fax: +81-744-29-8811; E-mail: hit3910@gmail.com

**Table S1. Correlations between changes in HbA1c, body weight, and random blood glucose, and those in DTR-QOL total/domain scores.**

|  | Correlation with change in HbA1c | | |  | Correlation with change in weight | | |  | Correlation with change in random blood glucose level | | |
| --- | --- | --- | --- | --- | --- | --- | --- | --- | --- | --- | --- |
| Domain | *n* | Correlation coefficient | *p*-value |  | *n* | Correlation coefficient | *p*-value |  | *n* | Correlation coefficient | *p*-value |
| D1. Burden on social activities and daily activities | 203 | −0.02 | 0.751 |  | 204 | −0.18 | 0.010 |  | 198 | 0.01 | 0.931 |
| D2. Anxiety and dissatisfaction with treatment | 200 | −0.10 | 0.168 |  | 201 | −0.20 | 0.006 |  | 195 | 0.05 | 0.522 |
| D3. Hypoglycemia | 199 | −0.03 | 0.716 |  | 200 | −0.22 | 0.002 |  | 194 | 0.05 | 0.463 |
| D4. Satisfaction with treatment | 201 | −0.22 | 0.002 |  | 202 | −0.05 | 0.505 |  | 196 | −0.05 | 0.454 |
| Total Score | 197 | −0.12 | 0.102 |  | 198 | −0.24 | < 0.001 |  | 192 | 0.03 | 0.702 |
| Correlation coefficients and *p*-values are based on the Spearman rank correlation coefficient. | | | | | | | | | | | |
